# The Replier Replied to, and the Reviewer Reviewed; a Letter to Lunsford P. Yandell, M.D., Containing Strictures and Thoughts on the Errors and False Doctrines of Chemico-Physiology

**Published:** 1844-07

**Authors:** Charles Caldwell


### WESTERN JOURNAL

OF

### MEDICINE AND SURGERY.

JULY, 1844.

Art. I.—*The Replier replied to, and the Reviewer reviewed*— A letter to Lunsford P. Yandell, M.D., containing Strictures and Thoughts on the errors and false doctrines of Chemico-Physiology. By Charles Caldwell, M.D.

Dear Sir—

This letter will consist chiefly of extracts from a much longer and more multifarious one, not yet published, but containing materials for a separate work, which may perhaps appear at some future period. Being therefore, in its structure, the product of an effort at literary *patch-work* (a form of book and essay-making in which I am neither practised nor skilled, and toward which I have neither predilection nor ambition) you will not I trust expect to find in it an ample exhibition of either compactness or neatness, order or symmetry. If you do, you will be disappointed; for, in the management of mental no less than of bodily materials, I am a very ordinary and every-day mechanist. In arranging and joining together the fragments destined to compose this communication, my principal aim will be, so to dispose them, as to render the production clearly intelligible, correct in fact and sound in argument, pertinent in matter, and direct in its bearing on the important end it is designed to subserve.

Your review of my Critique on Animal Chemistry, published in the December number (1843) of the Western Journal, is the original source of this letter. Had not that publication been introduced to my attention, this one would never have been obtruded on yours. Whatever therefore may be the issue, directly or indirectly, for good or for evil, of the article I am writing, will be, in a great measure, if not entirely, the fruit of your action. If valuable, yours will be much of the merit and credit; if the reverse, a corresponding share of the blame will be yours.

To apprize you of the immediate purport of my letter, and to enter on the business of it without further preface or delay, allow me to say, that I consider your review as not only a very extraordinary, but, *in several respects*, a very injudicious and *exceptionable* paper. Nor, in its objectionable qualities, does it present only a single aspect. Far from it; as a brief exposition of it will satisfactorily show.

It is exceptionable in its bearing on science; because, as will presently be made appear, it is replete with error, groundless representation, and false doctrine—exceptionable toward yourself, as a writer and reviewer, because it abounds in striking instances of self-inconsistencies and self-contradictions—toward my critique on Liebig, because the estimate it places on it, as a production of intellect, is far below that which it has received from every other publication, within my knowledge, in which it has been noticed, and no less, as I verily believe, and shall endeavor to show, below what justly comports with its merit—and objectionable toward myself in the following particulars.

It represents me as little else than one of the dullest and weakest minded of men, by denying to me the possession of a sufficient amount of reason and ability to frame and offer a single sound or "plausible objection" to any portion of Liebig's Animal Chemistry, and of penetration and sagacity even to *detect* the errors which actually exist in that publication.

It imputes to me the most consummate, persevering, and culpable negligence, as respects the study of physiology, and its necessary consequence, a corresponding degree of ignorance of that branch of science. That imputation, so fatal to my standing if true, it attempts to make good, by proclaiming me to have been *asleep toward physiology*, and all the improvements and discoveries made in it, DURING "THE LAST HALF CENTURY!!"

Lastly, if my ears have not deceived, or my memory betrayed me, or the two conspired to delude and mislead me, the tone, temper, and tenor of your review have been regarded with surprise, and deeply condemned, by every intelligent reader of it, of whose opinion respecting it I have heard an expression. And not a few such expressions have reached me—some of them very recently. Several of our common acquaintances have even seriously asked me, after a perusal of your review, whether there had not occurred between you and myself *an open quarrel?* And, on my replying in the negative, they expressed their *astonishment*, that, without such a cause, you had written such a paper. Nor did their sentiments of the subject differ from my own.

When I first looked into your review, I was actually amazed at the spirit of *apparent* unfriendliness, not to say *hostility*, which imbued and pervaded it—beginning on its *first* page and terminating on its *last*. And, that which on its face, was perhaps *only apparent*, seemed changed into *reality*, by certain forms of expression, the symbols of discourtesy, taunt, and disrespect, which, without any studied attempt at concealment, project from many of its pages, too much "like quills upon the fretful porcupine." To express myself on the subject summarily and significantly, no consideration short of a serious quarrel, or a determination to provoke one, could induce me to review in a manner so actually *unfair*, and so apparently *inimical*, a work from the pen of either of my colleagues—or in truth of any other respectable writer. The unfairness of the review, should a call to that effect be made on me from the proper source, I am prepared to demonstrate. But I cannot do so, on the present occasion, without an inadmissible protraction of this letter.

True; in a conversation on the subject, invited by yourself, you frankly disavowed the *premeditated design* to introduce into your review of my Critique, in substance or manner, spirit or language, any thing in the slightest degree offensive or exceptionable to me. And, for two reasons, I fully confide in the sincerity of your declaration—the guaranty of your veracity as a christian, and of your honor as a man; both of which were specially invoked in your disavowal—and the *fact*, that you had never experienced from me the shadow of a cause for an offensive act, or an exceptionable word.

Still, however, your review, with all its *seemingly exceptionable qualities*, is abroad in the land, through the instrumentality of the Western Journal; and its influence can be met and nullified by nothing short of a *public* disavowal, through the same work, and to the same effect, with that which you made privately and verbally to myself.

Of the several exceptionable features of your article, I have spoken somewhat fully in the manuscript, to which I have already alluded. But, for want of room, I cannot include the consideration of them all in this letter. Nor do I deem the measure, at present, either necessary or important.

Your own disavowal in the last number of the Journal, has extinguished the feature to which it relates. And the charge against me of my "*fifty year's sleep*," in relation to physiology, and its associate branches of knowledge, is so wild and extravagant, as to carry along with it, *in its utter improbability*, its refutation and overthrow. No one, even of the "*million*," who have only heard my name, will receive it with credit. Hence it is dismissed, for the present, as having no claim to further notice. Yet to exhibit, in still bolder relief, the enormity of its departure from fact, I say fearlessly, that, however humble my productions may have been, I have written and published, during the present century, on physiology and its connexions with chemistry and other branches of knowledge, more—much more than has ever been done by any other American—perhaps in the proportion of *three to one!* Hence I have assuredly been *awake* to it!

But two features then which demand my attention remain—your attempted disparagement of my Critique on Liebig—and the false doctrines you have endeavored to sustain. To them alone therefore shall my strictures be confined.

In testimony of the failure of your attack on my Critique to effectuate its purpose, I shall introduce the following extract from my manuscript.

Having already very slightly adverted to the fact, I now repeat, and shall prove my position, by an adduction of testimony, that, as far as my information in the case extends, not a single reviewer or writer, of standing and authority, in the United States, concurs with you in opinion, respecting the *distinguished* and *unqualified* merit of Liebig's chemico-physiology, and the *corresponding demerit* of my Critique on it. For, as regards the two productions, you deal in *wholesale terms*, pronouncing the former excellent nearly throughout, and the latter possessed of no excellence at all. I shall forthwith, however, present you with a different view of the matter, and evince to you that several writers of ability and eminence, on both sides of the Atlantic, whose names I shall cite, unite in sentiment with me, as regards the philosophy of Liebig, and are in direct opposition to you, in relation to the soundness and ability of both *his* writings and *mine*—condemning the *former*, and commending the *latter*. And of these, some are professed chemists, as well as physiologists.

Among the writers of the United States, I shall first refer to

Dr. Hare, the celebrated and veteran Professor of Philadelphia, one of the ablest and most accomplished chemists, both scientific and practical, that our own, or any other country has produced. Against some of the errors and faults of Liebig he has sternly protested, and spoken of them in terms as well as sentiments, not dissimilar to those which I have myself employed. To his writings therefore I am authorized to appeal, in support of the belief, that my Critique is by no means the weak and worthless production that you have pronounced it. Nothing resembling the writings of Professor Hare can be justly regarded as of a caste so degraded.

In his introductory lecture of last autumn (1843), that distinguished gentleman, after having bestowed, as I myself have not failed tc do, high commendation on the Professor of Giessen, on account of some portions of his writings, proclaims other parts of them to be, in his own strong and decided language, "in various instances, *bold*, *hasty*, *inconsiderate*, and *inaccurate*." And such is the very charge that I had previously preferred against them—a charge however of which Professor Hare was wholly uninformed.

Nor is this all that the illustrious chemist of Philadelphia has alleged in opposition to Professor Liebig. Far from it. In his further strictures on his writings, he likens that far-famed and ambitious innovator to a "military leader, who, after marching through a country with drums beating and colors flying, should have his triumphs loudly sounded, as if a complete conquest had been effected, while leaving behind him fortresses, of which the knowledge had prevented more cautious and considerate leaders from having previously undertaken the same expedition." Nor does he halt even here in his unsparing condemnation. In addition to the foregoing, he expressly declares that, in his own words again, "the philosopher of Giessen has deluded himself and the readers of his essays with the prospect of an elucidation of the mysteries of animal and vegetable physiology, *which it is beyond the present state of chemistry to afford.*" Ay; and let me add, beyond *any* state of chemistry; because those mysteries *pertain exclusively to another science—the science of life.*

Such are the expressions of Professor Hare, in relation to the philosophy and proceedings of Professor Liebig, and such their similarity to declarations in my Critique, of which you have spoken in terms of the most unqualified disparagement and disrespect. In truth your great leader in chemico-physiology has plunged into the grossest of blunders, from the same cause which has inflicted so serious an injury on your review of my Critique—*negligent investigation*, *over-hasty composition*, and *assertion beyond fact.*

When that celebrated chemist is in his laboratory, *performing experiments*, I have no doubt of his great ability, accuracy and skill. But, when in his study, engaged in his *speculations*, I have as little doubt of the unbridled wildness, extravagance, and fallacy, in which he indulges. His writings clearly evince, that he is alternately under the influence of the two opposite conditions of mind, calculated for the production of these two opposite results. Hence the glaring inconsistencies of his doctrines, and the mischief they portend to hasty readers and superficial thinkers, who neither calmly reflect, nor clearly discriminate.

Dr. Kemp, another highly respectable American chemist, has spoken with directness and decision against the hypothesis of Professor Liebig. Nor do I feel myself forbidden to add, that, on his late visit to this place, the Professor of Chemistry in Yale College, whose competency to decide on such subjects can hardly be surpassed, though he highly commended Professor Liebig, in some respects, spoke cautiously of him in others, and respectfully in all, did not however either conceal or in any way disguise his conviction, that, on several points he had asserted in his writings what he had not succeeded in verifying in his laboratory—and that, in so doing, he had not conformed to the canons of chemistry, as *an exact science.*

In condemnation of many errors and extravagancies in the works of Professor Liebig, Dr. Bird, an American physician, and a very able writer, cordially and earnestly unites with me, in the strictures and thoughts contained in my Critique on Animal Chemistry.

Were it not that the extract would add too much to the length of this letter, it would be gratifying to me to insert in it a passage or two from that author, that you might, with the less trouble, become acquainted with the striking similarity of his objections and mine, in both matter and manner, to one of the most extraordinary and ludicrous blunders, or wanton fabrications (for such blunder or fabrication it certainly is) that discredits the pages of medical literature. It is a bold and broad assertion to the following effect:

The carbonic acid gas, produced by the fermentation of unripe wine, in the stomach of a Dutchman, passes through the entire walls of that organ, consisting of mucous and cellular membranes, blood-vessels and nerves, muscles and peritoneal envelope—through the diaphragm, with its peritoneal and pleural linings on its lower and upper surfaces, through the substance of the lungs and their pleural covering, and, continuing its course, makes its way also through their mucous lining into their air-cells, and, by usurping and retaining possession of them, to the exclusion of atmospherical air, strangles, poisons, or by some other mode of action, takes away the life of the unfortunate Dutchman!!!

Such is circumstantially the story narrated by Liebig, and endorsed by yourself—for *substantially* you *have* endorsed it!! And if there be, in the wide scope of tale-telling, fable-framing, and wonder-weaving, in this age of fable, tale, and wonder, a single fiction that falls short of it, in *vraisemblance*, I know neither *where* it is, nor *what* it is. Nor do I believe in its existence. Yet have you, I say, through the influence of some unaccountable hallucination, virtually proclaimed and recorded your adhesion to it, in common with its author's other aberrations from sound fact, and sober probability.

In case of your being curious to peruse Dr. Bird's animadversions on this new "Legend of the Rhine," you may find them in Bell's "Bulletin of Medical Science," vol. i., July No.

p. 260. Introductory to the animadversions, moreover, you will find, in the same page, the editor's notice of their striking similarity to my own remarks on the same subject, in my "Physiology Vindicated."

But why should I any further trouble either you or myself with this *full-grown* air-penetrating and air-rambling story? since you have yourself procreated in *embryo*, one but little inferior to it, in all that awakens incredulity and excites surprise. In reply to some remarks, in my "Physiology Vindicated," on the respiratory apparatus of birds, you observe:

"This is easily answered. The permeability of the bones of birds by the atmosphere being admitted, the *chemist asks no more*." (Yes, but the *physiologist does.* He asks for a *specific organ* to perform a *specific function;* and from *nature* he always receives it. But you offer to him only a *common* organ to perform a *special* function, which he instinctively rejects; because nature commands him to reject it. He "asks for bread," that he may swallow and digest it, but "you give him a stone," which he can turn to no useful account. But to let you proceed with your tale). "He," (the chemist), "knows that the air, once in contact with the moist animal membrane, has no difficulty in reaching the circulation. It does not obviate the difficulty, denying that these air-tubes are part of the true respiratory apparatus. It is enough that oxygen finds its way into them."

Without taking the trouble, and spending the time requisite to analyze this extraordinary scrap of chemico-physiology, I shall briefly observe, what every one must perceive, that its object is to create a *substitute for the lungs*, in the momentous function of the *arterialization* and vitalization of the blood.

According to this *new school scheme* of yours, in cases of defective respiration, open and shut the mouth repeatedly, allowing atmospheric air to come into contact with the "moist animal membrane" that lines the cheeks and fauces, swallow it repeatedly with saliva, bringing it thus into contact with the moist membrane which lines the œsophagus and stomach —erase the cuticle extensively, so as to give it access to the moist cutis vera—or more especially, inject it copiously with a syringe *per anum*, and give it free admission to the moist membrane lining the large and small intestines—adopt, according to the exigency and character of the case, any or all of these expedients, to bring the atmosphere into contact with a "moist animal membrane"—and a fig for the lungs and their function!—the blood, especially as the *bones* are to aid in the process, will be arterialized and vitalized without them—and the other functions of the body will go healthily and cheerily on! Such is your scheme of respiration and the revivification of the blood, which, if carried out to its full length, *would be infinitely ludicrous.* But I can pursue it no further. Leaving it therefore to the keeping and tender mercies of others, I shall return to the topic, from which I have digressed.

Having finished all I mean to say, for the present, in relation to Americans, have the goodness to accompany me to Europe, that I may exhibit to you some of the testimony to be collected there, respecting the subject I am now considering. And here again, for the sake of the conciseness imposed on me, by considerations which I may not resist, instead of crowding my pages with extracts from books, I shall be compelled simply to refer, by name and place of residence, to writers, who have recorded their views of chemico-physiology.

In France, Dumas, Virey, and Boussingault, whose reputation and standing are too high, and too well known to need any commendation—in Ireland, Graves and Stokes, two of the ablest medical philosophers and writers of the day—and in England and Scotland, Pereira and others, in no way their inferiors—all these writers, whose works are their passports to distinction and authority in science and letters, have placed on record their sentiments of many of the chemico-physiological and pathological fancies of Liebig and his followers; and their condemnation and rejection of them are unanimous and unqualified. And of three of those writers in particular, Virey, Graves, and Pereira, I may now say the same that I have already said of Bird. They have selected from Animal Chemistry, for their strictures and disappròval, the very same errors and fallacies that I have myself selected, in Physiology Vindicated. Nor is this all. Their objections against those faults rest on the same ground; and, in their illustration and defence of that ground, they have not only adduced the same facts and matters of argument, but have employed them very much in the same way. In truth, though they and myself wrote about the same time, in different and remote quarters of the globe, and without the slightest suspicion of what each other were doing, we have notwithstanding, on sundry topics, written so strikingly alike, as almost to subject ourselves to the charge of mutual plagiarism. And that instinctive and unconcerted unanimity of sentiment alone, independently of other considerations, amounts to evidence of no ordinary validity, that we are correct in our views of the points contested, and our antagonist incorrect. Our common unconventional agreement with each other resembles the harmony of unperverted perception, judgment, and common sense with what is natural and true, and their antipathy to the reverse.

The facts, however, which have been hitherto adduced, are but *indirectly* evidential that my Critique on Animal Chemistry possesses much of either ability or truth. They show, indeed, its concurrence in fact, opinion, and argument, with other productions of acknowledged ability and value on the same subjects. But that amounts to nothing more than presumptive testimony, that its merit and value are in any measure equal to theirs. Permit me to trouble you with a few further facts and considerations testifying more immediately and expressly to the valuableness, in both matter and manner, of my "Vindication" of physiology from chemical adulteration. And here I am compelled to ask your indulgence in accompanying me in my return to our own country.

In every *respectable* and *authoritative* Medical Journal of the United States, I think, but *one*, my Critique has been noticed. And in each of them, the Western Journal excepted, it has received not mere approval, but *actual encomium*, sufficiently lofty to satisfy the craving of the most ambitious writer.

True; the veteran and well-disciplined editor of the Boston Medical and Surgical Journal, finds fault with what he considers an excess of severity and reprehensiveness in some of my strictures on the writings of Liebig, and the mistaken judgment of those who have inordinately overrated them. But, notwithstanding this, he pronounces the Critique a work marked by even "gigantic powers of mind"—and declares, that, had it not *been* for his "Ishmaelitish temper and inordinate ambition" its author might have been, "twenty years ago, a dictator in medicine in the United States." I have not, at this moment, the Boston periodical before me. But such *precisely* are the sentiments expressed in it, and such, very nearly, the language employed. If the encomium is too lofty, the fault is not mine. On the erring judgment and taste of the editor let the blame descend.

As respects its matter, argument, and manner, as well as its style, the able and accomplished editor of the New York Journal of Medicine speaks of my Critique in the language of panegyric. And no less complimentary to it have been his private letters to me.

Of Dr. Bell, of Philadelphia, decidedly one of the ablest and most impartial reviewers in America, the same is true. His notice of my Critique, in his Bulletin of Medical Science, (July No., 1843, p. 247), is quite eulogistical. A few of his remarks are as follows.

"But among all those who have stepped forward to do battle for the right, to defend physiology against the usurpation of one of her hand-maidens, chemistry, we will venture to say, that no one gives keener and more home thrusts than our veteran friend Dr. Caldwell.

"On the subject of animal heat the criticisms and corrections of Dr. Caldwell are numerous and cogent, based as they are on physiology, and amply illustrated by references to natural history and anthropology—the habits of animals and of man in opposite and contrasted climates. Even as a mere exercise of intellect, as a rich repast composed of stores of observation and learning, the essay before us merits careful, as it will secure, when once taken up, an eager perusal by the medical student, we care not whether he be a tyro, or one deeply read in his profession."

Would the celebrated editor of the "Select Medical Library" and of the "Bulletin of Medical Science," who has examined and reviewed, I believe, more medical works than any other man in the United States, who is proud of the reputation as a reviewer, which he has already achieved, anxious to retain, and ambitious to augment it—would a gentleman of this description thus applaud a work neither characterized by "general ability," nor containing either "one solid objection, or one "plausible argument," and whose author, in consequence of his negligence of "*fifty years*" toward its subject, is of necessity supremely ignorant of it—is it, in the nature of things, possible for an *anomaly* of this kind to occur? Put the question to *common sense* and experience, judgment, and *conscience*, and, from their reply, my Critique will have nothing to fear. Call you this the language of vanity and self-applause? I call it the award of PLAIN TRUTH,—and an enlightened and fair-minded community will concur with me in the significancy and correctness of the name.

In numerous letters received from correspondents, similar commendations have been cordially bestowed, on my "Physiology Vindicated." The following extract, in relation to that work, is from a private letter, by Professor Paine, of the Medical Department of the University of New York—one of the most erudite and able physiologists of the age—and I am permitted to publish it:

"The whole work "(Physiology Vindicated)" is admirable throughout. All the facts are pointed—the arguments perspicuous and forcible, and the conclusion irresisitble. What you say of the ability of the system to absorb and render latent a certain amount of the caloric of high surrounding temperature, as shown by the experiments of Fordyce, &c., is strictly original, and of great importance. I can at present see no mode of escaping your reasoning and conclusions."

I received also, but a few days ago, from one of the ablest medical philosophers on this side of the Atlantic, or indeed on either side of it, a letter, from which the following is an extract. After paying to me, on the ground of certain articles of mine, published in the three last numbers of the New York Journal of Medicine, a compliment, which I shall not here repeat, he proceeds to say:

"I see by the foreign journals, and by private letters, that Organic Chemistry is fast on the wane in Europe. It was one of those *convulsive paroxysms*, that, now and then, shake the moral world with omens of complete destruction; but which as speedily pass away, and, like the shock of an earthquake, leave things as they were before. Truth will soon shine with greater lustre; and therefore I never regret those violent tornadoes; however much I may have breasted myself to the storm. It is even pleasant to look back, and to feel that you are not yourself swept away by the hurricane, and that you had the daring to brave its fury. Who now remembers or cares for Liebig's new philosophy in all America? It seemeth but a dream to that multitude of enthusiasts, who worshiped without the slightest knowledge of the subject of their adoration. I never met with one who could tell me what it was that had called forth his admiration; and, when pushed for a reply, all, save one, confessed that they had never perused the work which called forth their idolatry! A reviewer, ardent in its praise, admitted that he could never get beyond the *four or five first pages!* So much for Liebig, the credulity, and fashion of the intellectual world!"

My own experience in the matter has been very much the same with that of my able and distinguished correspondent. Yourself perhaps excepted, I have never seen a *fairly ignited* admirer and votary of Liebig, who had even *read* his Animal Chemistry, (I mean the entire work), much less *studied* it. Yet if not carefully studied—much *more* carefully indeed than most persons will ever submit to the toil of doing, that work cannot possibly be understood, in the entire extent and depth of its *folly and extravagance.*

Nor have I ever yet seen a physician, who, however ardent and enthusiastic he might be in his praises of Liebig, was willing seriously to say, that he had derived, from the writings of that author, a single fact, or thought, or suggestion, in the slightest degree useful to him in either the science or the practice of his profession. Nor can such useful matter be found in them. If my memory deceive me not, I have heard, from your own lips, an express acknowledgment that his pathological and therapeutic speculations are *worthless.* Somewhat to strengthen your expression, I pronounce them *sheer balderdash!* And whatever vehemence or even bitterness of contradiction the *Faithful* may offer to the sentiment conveyed by these words, I defy their disproof of it. Nor do I stop here.

I further say, without hesitation, that, as concerns his notions of the functions of living organized matter in general; their causes, laws, and modes of performance, Liebig is but a meteor, which, in the night of their ignorance, flashes, attracts the gaze, and excites the admiration of the *many*, without, however, long misleading the senses of the *few*, and on the opening of the daylight of reflection and truth, like mist under sunshine, fades and disappears.

Such is the aggregate of all the testimony I have time and room to submit to your inspection, but not even of a moity of what might be easily adduced, in support of the belief, that my Critique on Liebig possesses much more ability and merit, as a specimen of science, and a work of intellect, than you have conceded to it. And, were your review now to be written, I confidently believe that one of the two following issues would occur. Either you would not write it at all—or you would embody in it more observance and courtesy of manner, and a higher estimate of the general character of my Critique, than you have done, in that which you have already written.

My reason for this opinion is a firm belief, founded on its general and obvious immaturity, that your review is a very hasty and careless performance; and that after having, more deliberately reflected on the subject, you would not again put your reputation at stake, by a work so far inferior to what you are able to produce. A remark or two more, and I shall be done with this topic.

I can hardly doubt your being somewhat startled, if not alarmed, at the formidable phalanx, against which you have indirectly waged war, by your inconsiderate attack on my "Physiology Vindicated." For, you must necessarily perceive that you are in actual conflict with every individual, whether reviewer or common author, who has either bestowed high commendation on my "Vindication," or assailed the notions of Liebig, on the same points, grounds, and principles, which I have assumed, and with nearly or quite the same facts and arguments that I have employed in that publication.

What further measures, in the controversy, either you or those individuals may adopt and pursue, I profess not to know; nor does it, at present, concern me to inquire. But this I know and openly declare. Were that able and distinguished corps of writers arrayed against me, in the contest, as they are against you, I should feel it due to myself, as well as to truth, either to fortify my position, by additional muniments, more solid and impregnable, than any you have yet thrown around yours—or retreat from it, as weak and untenable. And were I called on, as an umpire, in a case of the sort, such is the character of the decision I should urge. But my province, in the present discussion, is, to *establish* and *defend* true doctrines, rather than to offer counsel for their application and employment, as means toward an end.

Leaving you therefore for the present, unannoyed and uninfluenced by either advice or example, to the enjoyment of your own fancies, and to the execution of your own resolutions and measures, I shall pass to the consideration of other topics.

Having said more I fear than *you may think* the matter worth, and perhaps much more than it *really is* worth to the public or to posterity, in defence of the ability of my Critique, as a work of intellect, I shall now submit to you a few remarks on some of your own views and speculations, on certain special points of science. And here, to make, if possible, some amends for antecedent prolixity, I shall endeavor to be brief. The more certainly therefore to compass my design, it is my purpose, instead of discussing and refuting all the errors you have piled on each other, in your review, like Ossa on Pelion, and mount Olympus on that, to merely *mention* a few of them, and reserve for analysis and *refutation* only one of the most gigantic of them.

In your remarks on hyperborean and tropical savages, you immerse yourself in one of the most extraordinary concoctions of inconsistency, error, and self-contradiction I have ever witnessed. And, in the commission of those faults in logic and science, you are always governed by the exigencies of your cases. Your errors I mean, are very skillfully dovetailed into your cases; so as to make of the matter a well-finished and symmetrical job.

In one crisis of their condition and requirement, your *northern* tribes take, during their intensely cold weather, severe exercise in the open air, that they may inhale a sufficiency of oxygen, to burn up the abundant carbon of their gluttonous meals—and, because *they* have but little carbon in their systems, your *tropical* people seek breezy shades and dwellings, calm repose, and cooling beverages.

But, by a touch on your magical curtain-wire, the scene changes, and all concomitants and requirements change along with it. The arctic man now crams himself with whale-blubber, and, instead of taking exercise in the open air, snores away his time, during the immense severity of the winter, in his air-tight and comfortable ice-hut—crowded by his family and associates, and filled with semi-stagnant air adulterated by the exhalation from their lungs and persons; and the tropical man exercises laboriously, in the midst of a highly heated atmosphere, and under the very path of an unclouded and fiery sun.

At one time your polar inhabitant, that he may be sufficiently supplied with fuel for his body-stove, cannot subsist except on animal food containing in its composition *eighty-hundredths* of carbon; and, at another, for the same reason, a portion of starch, or some other form of vegetable food, containing but *forty-four-hundredths* of carbon, is alike indispensable to him. In the former case, to become duly carbonized, he must swallow an immense quantity of carbon; and in the latter, for the same purpose, it is equally essential that he use food containing but little more than half the amount of that article!! And thus alternately, concomitantly, or consecutively, according to the veering wants of your hypothesis, (not of your savage tribes) its changing but ever-accommodating requirements, by some wizard influence, are brought to its aid. If these statements, or any others I may offer, be denied or contradicted, I am prepared to make them good *by an adduction of facts.*

Your notion that the temperature of infants is higher, under like circumstances, than that of adults, is so utterly groundless and improbable, that its being seriously entertained or even imagined by any observing and reflecting man, is positively astonishing. By every fact in nature that bears on it, its fallacy is demonstrated.

No less in opposition to reality is your misguided fancy that living trees have no inherent power to maintain, each its own appropriate temperature. To every one who has skilfully experimented and attentively observed on the subject, it is perfectly well known, that vegetables in general (*trees* not excepted) possess a *heat-regulating* power, as certainly, but not so strikingly as animals. And those who have neither observed nor experimented in the matter, as I positively believe is the case with yourself, have no just ground for any *real opinion* about it. All that such persons know, or rather *pretend* to know, in relation to it, is *supposition*, *hearsay*, or *unenlightened imagination*.

Under this head may be noticed your singular incredulity, no less singularly expressed, as to the heat-preserving property of living vegetable seeds. To *living wheat* this incredulity is expressly directed. For instruction on this point, allow me to refer you to any agriculturist of observation and intelligence in the country, who cultivates the wheat plant on a large scale. An individual thus prepared, will promptly assure you, that, if you shut up, in the winter season, in a close granary, a few hundred bushels of *dry* living wheat, *in bulk*, the temperature of the room will be maintained by it, several degrees above that of the atmosphere, in empty adjoining rooms, and of that out of doors. And such an individual has no theory on the subject either to establish or support. He deals only in facts.

It cannot be unknown to you that a certain degree of temperature is essential to the vitality of everything that lives. Sink the temperature below that degree, and the issue, in a given time, is *certain death.* It would be an anomaly then, in the wise and beneficent work of creation, were a *practical instinct*, acting, when necessary, toward the preservation of the requisite temperature, denied to any sort of living matter. Man, in this respect, holds his life, on the same principles, and by the same tenure, with the grass he treads on, and the wheat on which he subsists. To him a given temperature is indispensable to life; and he has a power to preserve it. Nor is a given temperature, I say, less essential to the herbage of the field, and the seed, from which it springs. Wherefore then should not they be endowed with a like power, as far as they need it for the purposes of existence, by the GREAT AND COMMON PARENT OF ALL? without whose notice, and agency through his laws, not a sparrow falls, a seed perishes, nor a leaflet withers. The *perfections of the Deity*, from all we know of them, resolve this question, and testify to the truth of the position I am maintaining. Consult on this topic the very tenets and principles of the religion you profess; and, if correctly interpreted, they will instruct you much more correctly and thoroughly than erring man can do. And their instructions will be to the same effect with that which I have attempted to impart.

Of your remarks on the brain of a person starved to death, conceived and offered as they are in a spirit of apparent taunt and disrespect, I shall only say, that they are weak and vulnerable in every point. Though I may not dwell on them now, I am prepared to show, on any convenient occasion, that they have nothing of either force or ingenuity in them. On the contrary, they are as deficient in intellectual soundness and efficiency of matter and argument as they seemingly are in courteous observance and respectfulness of manner.

To my statement, made in my Critique, that, in many patients, whose respiration is greatly impeded and limited, by tubercular disease, and other lung and thoracic affections, the temperature is, notwithstanding, *highly febrile*, you calmly reply, as if with ex-cathedral authority, that, in such cases, "the breathing, though limited, is much *more frequent*" than in health—or than in other forms of disease, where the lungs are not deranged.

This reply from *you*, who have practised medicine for many years, amazes me. In direct contradiction of it, I could state to you scores of cases, which have fallen under my own observation, but shall content myself with one, which alone is all-sufficient for my purpose.

It is recorded, on the authority of Graves and Stokes of Dublin, and appears in the 5th volume of the Dublin Hospital Reports. It is also referred to in the American Journal of Medicine, vol. 8, year 1831, p. 218, and is as follows:

Each lobe of the lungs was extensively tuberculated; the pulse beat 126 times in a minute; and the temperature was *highly febrile.* Yet did the patient inspire but 14 times in a minute! Look into the book of nature, that portion of it, I mean, called the *sick-bed*, and many analogous cases, I say, will present themselves to you.

I shall only add, that the patient here referred to was an adult, whose healthy inspirations by the minute, were, we may presume, about 19. Reconcile this case with your hypothesis, as best you may. And let it never be forgotten, that one *real* fact is as good as a thousand; because nature never contradicts herself.

Though the project is really too ludicrous to be treated seriously, I must not, on account of what I might, in every-day language, call the "*fun of the thing*" pass unnoticed your and Professor Liebig's scheme of manufacturing Esquimaux, Samoyedes, and other varieties of the Tartar race. It is to convert into hunters or fishermen, in a cold climate, Dutchmen, Frenchmen, Englishmen, or persons of any other diversity of the Caucasian family, making them go half-naked, to sharpen their appetites, and gorge them abundantly with whale-blubber, fat seal-meat, bear-meat or pork, and tallow candles, and furnish them, for their drink, with train-oil, or melted hog's lard—and the work is done.

Let them persevere in this course long enough (but you have not told us *how* long) and those who entered it, in the capacity of decent and temperate Caucasians, will come out of it, in appetite and habit, debased, gluttonous, and beastly Tartars. Instead of a pound and a half, or two pounds of animal, and a corresponding amount of vegetable food, each of them will now be able to eat and digest daily, and will have an appetite craving them, ten, fifteen, or even twenty pounds of whale-blubber, or of the fattest bear or hog meat, with a pound or two of tallow candles, and to drink a gallon or six quarts of train-oil or brandy, "without injury!!"

Such is the doctrine you must adopt and defend, or desert the banner and service of Liebig. And it is hard to say, which is most nauseous and difficult to be swallowed and digested—the German doctrine—or the Tartar meal!! I can master neither; and must therefore leave to men of more indomitable stomachs of mind as well as of body, less squeamish appetites, and more ostrich-like digestion, the tough, greasy, and repulsive concern!!

Could I condescend to an analysis of this narrative, *on physiologcal principles*, I could make such an exposure of it, as ought to make Professor Liebig, should the production ever meet his eye, to *blush to his shoulder-blades*, at being either the author or the propagator of a *blunder* or a *fiction* (whichever it may be) so shamefully untrue. As well might he assert, that it is customary for the *headless trunks* of German criminals, when executed on the block, to spring from the platform, and join the headsman in a *waltz* or a *rigadoon!!*

Once more. Your narrative of my experiment, in the ignition of charcoal by nitric acid, appears much more of a home-stroke by you, than any other that you have dealt in the contest. But its successfulness is *only apparent*. Had you given faithfully the details of the experiment, its aspect and language would have been very different from those you have made it present and utter. But, like many other witnesses, whose memories are not trust-worthy, you have forgotten—certainly you have *omitted* the only fact in the case, of any real moment in the present discussion. I allude to the *temperature* of the acid and charcoal, which I used on the occasion. And be assured, as I have *always stated*, that it was *far above* that of the warmest of warm-blood animals, and still much further above that of cold-blooded ones. As far as my memory now serves me, it was at about 150° of Fahrenheit. Yet have you asserted (Western Journal, Dec. No., p. 459) that the experiment was performed by me, and "carbonic acid" produced "out of the animal body, at a temperature *by many degrees lower*" than that of warm-blooded—and virtually indeed also of *cold-blooded* animals. For, in the passage of my Critique, on which you were commenting, I specially referred to the *latter class* of animals, as well as to the *former*. I do not assert that cold charcoal cannot be ignited by cold nitric acid. But I do assert that I never saw it done—though I have often both made and witnessed the trial. And I further assert, that when the articles were cold, or even *cool*, the late Professor Woodhouse always failed to ignite the coal, but usually, I believe *uniformly* succeeded, when they were hot. To yourself and your readers I commit this narrative without annotation. It is its own most clear and significant annotator. And it emphatically proclaims, that, though your narrative of the experiment meets exactly the requisition of your hypothesis, it very unaccountably fails to notice *the most important feature of the case.*

On the most signal, as well as the most important of your failures, I shall now submit to you the remarks, with which I design to close this letter, protracted already far beyond the limits originally assigned to it. My allusion is to your attempt, very confidently made, to explain, on chemical principles, the result of the long and far-famed experiments, performed in London, toward the close of the last century, by Messrs. Fordyce, Banks, and Blagden. Nor is my design to speak either too strongly or too disparagingly of that attempt, when I say, that I perceive in it, from beginning to end, but little, if any thing else, than what I am compelled to regard as an unbroken tissue of errors and mistakes—fallacies and failures. That this estimate of your effort may be the more clearly seen to rest on substantial ground, and to be sustained by indubitable testimony, I shall first state the leading facts of the case, as succinctly and clearly as possible, and then offer on them a brief and, I think, an unprejudiced and fairly-conducted comment.

This however must be done at a subsequent period; and in the mean time I submit to your consideration the following observations.

If I understand his doctrine correctly, the chemico-physiologist contends, that the production of animal temperature is always and *necessarily* accompanied by a simultaneous production of carbonic acid—and that the two processes and their products are equal to each other in amount—if the temperature be high, the acid is abundant—and the converse.

Let the truth of this doctrine be tested by the case of an incubated egg. After vital action has fairly commenced in that body, vital heat is known to be evolved in it. In proof of this, let two eggs, one of them living and the other dead, be incubated, in the same nest, by the same hen. While the animal sits on them, and communicates to them the heat of her body, their temperature is the same. But, the weather being cool, let her be removed from them, for an hour, or even less, and the temperature of the sound and living egg becomes the highest.

Of this change the reason is plain. A never-failing effect of vital action is the production of vital temperature. But, during the absence of the hen, vital action in the living egg continues. So therefore does the evolution of vital heat. But the dead egg, having within it, as a source of temperature, no vital action, loses the heat it had received from the hen, and becomes cool.

What is the fact however (I ask for information) as respects the production of carbonic acid? The question is put to the chemico-physiologist, who ought to be prepared to render an answer, his hypothesis being vitally concerned in the issue. Is either carbonic acid or water, or both, actually formed in the living incubated egg? If so, what are the evidences of the fact? Is the acid alone, the water alone, or a mixture of the two found within the egg, in the *folliculus aeris*, or elsewhere? Or do they escape through the pores of the shell? And are they detected without that covering?

These questions the chemico-vitalist ought to be able to answer, on the authority of experiment. And to me, an answer, thus sanctioned, whether it be *affirmative* or *negative*, will be gratifying—because I am in quest of sound knouwledge—not of conjecture or supposition—nor of mere victory in discussion.

I do not say that carbonic acid and water are not formed in the incubated egg. Do you, in behalf of chemico-vitality know and assert that they are? I need hardly add, that, in case neither of them be formed, the issue, as far as a single instance may avail, is in direct opposition to the hypothesis you maintain. Again.

During cold weather, take two eggs, one of them fresh and living, and the other stale and dead. Expose them, by the side of each other, to the same atmosphere; and, having suffered them to lie there for an hour, break them, and ascertain the temperature of their contents.

In this experiment, you will find the living egg to be perceptibly the warmest. Possibly the contents of the dead egg may be frozen, and its shell ruptured; while those of the living one are unchanged.

In this case, is any carbonic acid formed by the living egg? The question, being one involving science, is worthy of a direct answer, deduced from experiment. And I trust the answer will not be withheld. It ought, moreover, to be rendered by Professor Liebig, or some of his adherents, to whose hypothesis of animal temperature it specially pertains.

June, 1844.

